# Bonding Efficacy of Universal Adhesives to Fluorotic Enamel after Pre-conditioning with EDTA

**DOI:** 10.3290/j.jad.b2701635

**Published:** 2022-03-01

**Authors:** Fabiana Suelen Figuerêdo de Siqueira, Luana Paraíso Muniz, Lívia Câmara de Carvalho Galvão, Michel Wendilnger Cantanhede Ferreira, Alessandra Reis, Andres Felipe Millan Cardenas, Alessandro D. Loguercio

**Affiliations:** a Professor, Department of Postgraduate Program in Dentistry, CEUMA University, São Luis, Maranhão, Brazil. Research idea, designed testing assembly, performed bond strength experiments, co-wrote paper, proofread the manuscript.; b PhD Student, Department of Postgraduate Program in Dentistry, CEUMA University, São Luis, Maranhão, Brazil. Designed testing assembly, performed bond strength experiments, co-wrote paper.; c Professor, Department of Postgraduate Program in Dentistry, CEUMA University, São Luis, Maranhão, Brazil. Designed testing assembly, performed bond strength experiments, proofread the manuscript.; d PhD Student, Department of Restorative Dentistry, State University of Ponta Grossa, Ponta Grossa, Paraná, Brazil. Designed testing assembly, performed scanning electron microscopy, co-wrote paper, proofread the manuscript.; e Professor, Department of Restorative Dentistry, State University of Ponta Grossa, Ponta Grossa, Paraná, Brazil. Co-wrote paper, provided consulting for statistical analysis, contributed substantially to the discussion, proofread the manuscript.; f Professor, Department of Postgraduate Program in Dentistry, CEUMA University, São Luis, Maranhão, Brazil. Research idea, designed testing assembly, performed raman spectroscopy, co-wrote paper, proofread the manuscript; g Professor, Department of Restorative Dentistry, State University of Ponta Grossa, Ponta Grossa, Paraná, Brazil. Provided consulting for statistical analysis, contributed substantially to the discussion, proofread the manuscript.

**Keywords:** universal adhesives, ethylenediaminetetraacetic acid, fluorosis, dental bonding

## Abstract

**Purpose:**

To compare the effect of active pre-conditioning with 17% ethylenediaminetetraacetic acid (EDTA) vs 37% phosphoric acid (PA) on the resin-enamel microshear bond strength (μSBS), enamel-etching pattern, and in situ degree of conversion (in situ DC) of four universal adhesives on sound and fluorotic enamel.

**Material and Methods:**

In this study, 448 extracted human molars (224 without fluorosis and 224 with fluorosis) were sectioned into four parts and divided into 16 experimental groups based on the enamel surface (sound or fluorotic enamel), adhesive (Clearfil Universal Bond [CUB], Futurabond U [FBU], iBond Universal [IBU], or Scotchbond Universal [SBU]), and enamel conditioning agent (PA or EDTA). The specimens were stored for 24 h and tested under shear stress at 1.0 mm/min to determine the μSBS. The adhesive-enamel interfaces were evaluated for in situ DC using micro-Raman spectroscopy. The enamel-etching pattern was evaluated using a scanning electron microscope. The µSBS and in situ DC data were analyzed separately using three-way ANOVA and Tukey’s post-hoc test (α = 0.05).

**Results:**

Sound enamel showed higher μSBS and in situ DC compared to fluorotic enamel (p < 0.05). However, no significant difference was observed for μSBS, in situ DC (p > 0.05), or etching patterns when PA and EDTA etching were compared in sound and fluorotic enamel. Moreover, CUB and SBU showed higher mean μSBS than did FBU and IBU in both sound and fluorotic enamel (p < 0.05).

**Conclusions:**

Compared to PA, active pre-conditioning with EDTA showed similar μSBS and enamel etching patterns for all the adhesives in fluorotic enamel, without compromising the in situ DC.

Previously, the ingestion of fluorides constituted the cornerstone of caries prevention worldwide.^15^ It was believed that when the ingested fluoride was incorporated into the enamel, it made the enamel more resistant to the caries process.^21,56^ However, it has now been recognized that the effect of water fluoridation is mainly local and post-eruptive.^13,35^ In fact, chronic systemic ingestion of fluoride during tooth development can result in dental fluorosis.^13,35^

Dental fluorosis is a premature mineralization of the external enamel, which makes the sub-surface enamel increasingly porous and hypomineralized.^3,20,21^ Clinically, dental fluorosis compromises the esthetics of the teeth, ranging from narrow white lines to white opaque areas on the enamel surface, depending on the severity.^1^ In some patients, the enamel may become so porous that the outer layers break down, and the exposed porous sub-surface becomes discolored^22^ from light to dark brown.^27^

It is known that dental fluorosis may have considerable psychosocial effects on many patients and negatively affect their quality of life.^11,14^ Thus, several minimal interventions have been proposed to treat fluorotic enamel.^1,36,42,51^ One of the most useful approaches to improve esthetics is employing direct restorative procedures.^6^

However, the success of direct restoration depends largely on the micromechanical interlocking of the adhesive to the enamel.^65^ Due to the structural differences between fluorotic and sound enamel,^19,61^ bonding to the former is a clinical challenge, thereby compromising the clinical success of composite restorations.^9,54,67^ Several alternatives have been consider to improve bonding to fluorotic enamel, such as bur-roughening^17^ or active and prolonged application of adhesive.^9,54^

However, a simple alternative proposed to improve bonding to fluorotic enamel is the etch-and-rinse (ER) strategy with phosphoric acid (PA)^9,17,54,67^ instead of the self-etch (SE) strategy.^9,18,54^ Although ER could be considered a gold standard with respect to enamel bonding, it has been shown to be unsuitable for the dentinal substrate,^25,60,62^ because accidental dentinal etching may occur during the enamel-etching process, especially when a low-viscosity etchant is used. The effect of intentionally etching dentin with PA prior to the application of SE adhesives has been studied,^16,25,44,55,62^ and the results are conflicting and material dependent.

The demand for materials that are simpler and less technique sensitive has recently prompted manufacturers to develop “universal” or “multi-mode” adhesives.^37,46^ They are essentially one-step self-etch adhesives and provide dentists with the choice of selecting the adhesion strategy (ER, SE, or an alternative, ie, “selective enamel etching”).^39^ As was found for previous adhesive generations, the bond strength of universal adhesives to fluorotic enamel was lower than to sound enamel.^9,54^

Thus, different approaches that promote the effectiveness of the bonding of universal adhesives to fluorotic enamel need to be evaluated. One of the approaches used in sound enamel is pre-conditioning with ethylenediaminetetraacetic acid (EDTA),^32,33^ a chelating agent that promotes demineralization of the dental structure.^29,32^ This mechanism is most likely responsible for the increase in bond strength of SE adhesives in sound enamel.^32,33^

However, to the best of our knowledge, no study has evaluated whether pre-conditioning with EDTA improves the bonding efficacy of universal adhesives to fluorotic enamel. Therefore, this study compared the effect of active pre-conditioning with 17% EDTA vs 37% PA on the resin-enamel microshear bond strength (μSBS), enamel-etching pattern, and in situ degree of conversion (in situ DC) of four universal adhesives on bonding to sound and fluorotic enamel. The following null hypotheses were tested: pre-conditioning with 17% EDTA would not influence the (1) μSBS, (2) enamel-etching pattern, or (3) in situ DC of universal adhesives on sound enamel and fluorotic enamel when compared with 37% PA.

## Materials and Methods

### Tooth Selection and Preparation

This study used 448 extracted maxillary and mandibular caries-free human molars. The teeth used were classified according to the Thylstrup and Fejerskov (TFI) index to diagnose dental fluorosis according to severity.^47^ Before selection of teeth, two examiners were calibrated as described by Ermis et al.^17^

This study used 224 teeth with a TFI score of 0 (without fluorosis) and 224 teeth with a TFI score of 4 from individuals living in the endemic fluorosis areas of Ecuador (Chimborazo City) with 2 ppm fluoride in drinking water.^4,7^ Informed consent was obtained from the individuals under a protocol approved by the Ethics Committee Review Board (#3.183.007) before collecting the teeth. The teeth were disinfected in 0.5% chloramine, stored at 4°C in distilled water, and used within 6 months of extraction.

The roots of all teeth were removed by sectioning at the cementoenamel junction. Following this, 128 teeth were used to evaluate the μSBS (n = 8 teeth per group), 128 teeth were used to evaluate in situ DC (n = 8 teeth per group) at the resin-enamel interfaces, and 192 teeth (n = 8 teeth per group) were used to evaluate the etching pattern. Each dental crown was then sectioned diagonally across the long axis of the tooth to produce four enamel specimens (buccal, lingual, and two proximal) with an area of approximately 5 mm^2^.^30^

### Experimental Design

The teeth were then randomly assigned to 16 experimental groups according to the combination of the independent variables: (1) enamel surface: sound enamel and fluorotic enamel; (2) adhesive: Clearfil Universal Bond (CUB) (Kuraray Noritake; Tokyo, Japan), Futurabond U (FBU) (VOCO; Cuxhaven, Germany), iBond Universal (IBU) (Heraeus Kulzer; Hanau, Germany), and Scotchbond Universal (SBU) (3M Oral Care; St Paul, MN, USA); (3) enamel treatment: 37% PA (Condac 37%, FGM; Joinville, SC, Brazil; PA etch-and-rinse) as the control and 17% EDTA (Biodinâmica; Ibiporã, PR, Brazil; EDTA etch-and-rinse). For enamel etching pattern, a control group with no treatment was added. Randomization of the teeth for all testing was performed for each substrate (sound enamel and fluorotic enamel). A person not involved in the research protocol performed this procedure using computer-generated numbers.

### Sample Size Calculation

The main outcome of the present study was enamel bond strength. The mean bond strengths of universal adhesives obtained with PA etch-and-rinse conditioning of the enamel was considered in the sample size calculation.^30^ According to the literature, the mean bond strength (± standard deviation) of several universal adhesives evaluated was 20.4 ± 3.0 MPa. In order to detect a difference of 8 MPa among the tested groups, using α = 0.05, a power of 80%, and a two-sided test, the estimated minimal sample size was 8 teeth per group. The same number of teeth per group (n = 8) were used for the other tests (in situ DC at the resin-enamel interfaces and etching pattern).

### Microshear Bond Strength Test

All specimens from one tooth were embedded together in a polyvinyl chloride tube, 10 mm in height and 13 mm in diameter, using a chemically curing acrylic resin (Jet Clássico; São Paulo, SP, Brazil), leaving the enamel surface exposed at the top of the cylinder. The protocol suggested by Shimaoka et al^53^ was used to isolate the bonding area. Five to six perforations with an internal diameter of 0.8 mm were made in an acid-resistant, double-faced adhesive tape (Adelbras Ind e Com Adesivos; São Paulo, SP, Brazil) that was adapted to the enamel surface. This procedure was performed using a hygienic Ainsworth-style rubber-dam punch (Coltene Whaledent; Altstätten, Switzerland). The number of perforations on each enamel surface depended on the dimensions of the enamel specimens.

The universal adhesives were applied to the enamel surface in accordance with the manufacturer’s instructions, as described in Table 1. A single operator performed all bonding procedures as follows:

**Table 1:** Adhesive (batch number), composition, and application mode of the adhesive systems under different experimental conditions

Adhesive (batch number)	Composition*	Application mode*	
		PA etch-and-rinse (PA mode)	EDTA etch-and-rinse (EDTA mode)
Clearfil Universal bond (CUB) (700018)	HEMA, MDP, bis-GMA, ethanol, camphorquinone, hydrophilic aliphatic dimethacrylate, silane coupling agent, colloidal silica, water, and accelerators	Apply etchant for 30 s Rinse thoroughly Dry Apply adhesive and rub in for 20 s Dry by blowing mild air for 5 s Light cure for 10 s at 1200 mW/cm^2^	Apply 17% EDTA and rub in for 30 s Rinse thoroughly for 15 s Dry Apply adhesive as for the PA mode
Futurabond U (FBU) (1705288)	Liquid 1: Acidic adhesive monomer HEMA, bis-GMA, HEDMA, UDMA catalyst Liquid 2: Ethanol initiator, catalyst	Apply etchant for 30 s Rinse for 10 s Air dry 2 s Apply adhesive as for the self-etch mode Apply the adhesive to the entire preparation with a microbrush and rub in for 20 s. If necessary, re-wet the disposable applicator during treatment Direct a gentle stream of air over the liquid for about 5 s until it no longer moves, and the solvent is evaporated completely Light cure for 10 s at 1200 mW/cm^2^	Apply 17% EDTA and rub in for 30 s Rinse thoroughly for 15 s Dry Apply adhesive as for the PA mode
iBond Universal (IBU) (010024)	Acetone, UDMA, TEG-DMA, 4-methacryloxyethyltrimellitic anhydride, photoinitiator	Apply etchant for 30 s Rinse for 10 s Dry Apply the adhesive to the entire preparation with a microbrush and rub in for 20 s. If necessary, re-wet the disposable applicator during treatment Direct a gentle stream of air over the liquid for about 5 s until it no longer moves, and the solvent is evaporated completely Light cure for 10 s at 1200 mW/cm^2^	Apply 17% EDTA and rub in for 30 s Rinse thoroughly Dry Apply adhesive as for the PA mode
Scotchbond Universal adhesive (SBU) (638367)	MDP phosphate monomer, dimethacrylate resins, bis-GMA, HEMA, methacrylate modified, polyalkenoic acid copolymer, camphorquinone, filler, ethanol, water, initiators, and silane	Apply etchant for 30 s Rinse for 10 s Air dry 2 s Apply the adhesive to the entire preparation with a microbrush and rub it for 20 s. If necessary, re-wet the disposable applicator during treatment Direct a gentle stream of air over the liquid for about 5 s until it no longer moves, and the solvent is evaporated completely Light cure for 10 s at 1200 mW/cm^2^	Apply 17% EDTA and rub in for 30 s Rinse thoroughly Dry Apply adhesive as for the self-etch mode

HEMA: 2-hydroxyethyl methacrylate; MDP: methacryloyloxydecyl dihydrogen phosphate; bis-GMA: bisphenolglycidyl methacrylate; UDMA: urethane methacrylate; HEDMA: hexamethylenedimethacrylate; EDTA: ethylenediaminetetraacetic acid; PA: phosphoric acid; TEG-DMA: triethylene glycol dimethacrylate. * As per manufacturer’s instructions.

- PA etch-and-rinse conditioning (control group): Before adhesive application, the enamel surface was etched with 37% PA gel for the time recommended by each manufacturer. The surface was then water rinsed with an air-water syringe for 15 s. The adhesive was applied, and the manual pressure exerted on the microbrush (Microbrush International; Grafton, WI, USA) during application was equivalent to 35 g (Table 1).^31,45^
- EDTA etch-and-rinse conditioning: Before adhesive application, the enamel surface was actively etched with 17% EDTA applied on the enamel surface for 30 s.^33^ The surface was then rinsed with an air-water syringe for 15 s, followed by adhesive application as described for PA.

After application of the adhesive, transparent polyethylene Tygon tubes (Tygon Medical Tubing Formulations 54-HL, Saint Gobain Performance Plastics; Akron, OH, USA), with the same internal diameter as the perforations and a height of 0.5 mm, were positioned on the perforations over the double-faced tape, ensuring that their lumen were congruent with the circular areas exposed by the perforations. Resin composite (Opallis, FGM) was carefully packed inside each tube, and a clear Mylar matrix strip was placed over the filled Tygon tube and pressed gently into place. The resin composite was light cured for 20 s using a light-emitting diode light-curing unit unit at 1200 mW/cm^2^ (Radii-cal, SDI; Bayswater, Victoria, Australia). A radiometer (Demetron LED Radiometer, Kerr Sybron Dental Specialties; Middleton, WI, USA) was used to check the light intensity for every five specimens. These procedures were carried out using 10X magnifying loupes and with controlled temperature (23°C ± 2°C) and humidity (50% ± 5% relative humidity).^5^

After storing the specimens in distilled water for 24 h at 37°C, the Tygon tubes and the double-faced adhesive tape were carefully removed using a blade to expose the composite cylinders. Each specimen was examined under a stereomicroscope at 10X magnification. The bonded cylinder was discarded if there was any evidence of porosities or gaps at the interface.^38^

The specimens were attached to a shear-testing fixture (Odeme Biotechnology; Joaçaba, SC, Brazil) and positioned in a universal testing machine (Instron; Enfield, CT, USA). A thin wire (0.2 mm diameter) was looped around the base of each composite cylinder. The wire contacted the composite resin cylinder along half of its circumference. The setup of resin-enamel interface, wire loop, and center of the load cell was maintained in alignment to ensure the correct orientation of the shear forces.^52^ The crosshead speed was set at 1 mm/min until failure.

The μSBS (MPa) was calculated by dividing the load at failure by the surface area (mm^2^). After testing, the specimens were examined under an optical microscope (SZH-131, Olympus; Tokyo, Japan) at 100X magnification to define the location of bond failure. The type of failure was determined based on the percentage of substrate-free material: adhesive/mixed (A/M) failure at the resin/enamel interface or failure at the resin/enamel interface with partial cohesive failure of the neighboring substrates; cohesive (CE) failure exclusively within the enamel or failure exclusively within resin composite (CR); pre-test failures (PF).

### Enamel-etching Pattern

The etching pattern (n = 8 teeth per group) of the enamel surface was evaluated using an SEM (Vega 3 Tescan, Shimadzu; Tokyo, Japan). After teeth were prepared as previously described, the enamel surface for each type of enamel substrate (96 specimens of sound and 96 specimens of fluorotic enamel) was randomly assigned to be tested according to each group.

For this purpose, in the PA and EDTA groups, enamel specimens with an area of approximately 5 mm^2^ were previously etched according to the different experimental conditions (Table 1), rinsed for 15 s, air dried, the adhesives were applied (Table 1) but not light cured.

The enamel surfaces were immediately stored in acetone for 24 h to dissolve the monomer resins on the enamel surface.^43^ The specimens were then rinsed in deionized water for 5 min, immersed in a 96% alcohol bath for 5 min, followed by deionized water again for 5 min to dissolve and remove the SE primer and adhesive resins.^43^ Eight specimens of each substrate were used to evaluate the unetched enamel surfaces constituted the control group. All specimens were dried and dehydrated in a desiccator for 12 h, and the conditioned enamel surfaces were sputter-coated with gold/palladium in a vacuum evaporator (SCD 050, Balzers; Schaan, Liechtenstein). The entire surface of the treated enamel was examined using SEM (Vega 3 Tescan, Shimadzu). Photomicrographs of the representative surface areas were captured at 5000X magnification by a technician responsible for the SEM, who was blinded to all adhesive procedures.

### In Situ Degree of Conversion

The in situ DC (n = 8 teeth per group) was evaluated as per the protocol outlined by Cardenas et al^10^ and Loguercio et al.^30^ Enamel specimens with areas of approximately 5 mm^2^ were prepared as previously described. After that, the enamel specimens for each enamel substrate (96 specimens of sound and 96 specimens of fluorotic enamel) were randomly assigned for testing according to each group (12 enamel surfaces per group). After adhesive application, composite resin buildups were constructed on the bonded enamel using the same materials and protocols described for the μSBS test. After storage of the restored teeth in distilled water at 37°C for 24 h, the resin-enamel specimens were longitudinally sectioned across the bonded interface with a low-speed diamond saw (Isomet, Buehler) to obtain three resin-enamel slices.

The resin-enamel slices were wet polished with 1500-, 2000-, and 2500-grit silicon carbide paper for 15 s each. They were then ultrasonically cleaned for 20 min in distilled water and stored in water for 24 h at 37°C. The micro-Raman microscope (XploRA ONE Raman microscope, Horiba Scientific; Piscataway, NJ, USA) was first calibrated for zero and then for coefficient values using a silicon sample. The samples were analyzed using a 638-nm diode laser with a 100X air objective. The Raman signal was acquired using 600 lines/mm grating centered between 500 and 2000 cm^-1^, and the employed parameters were 100 mW, accumulation time 30 s, with 5 co-additions, spatial resolution 3 µm, and spectral resolution 5 cm^-1^.

Spectra were captured at the resin-enamel adhesive interface at three random sites for each specimen. Post-processing of the spectra was performed using LabSpec 6 Spectroscopy suite software. Additionally, the spectra of uncured adhesives were used as references. The ratio of the double-bond content of monomer to polymer in the adhesive was quantified by calculating the ratio derived from the aliphatic C=C (vinyl) absorption (1638 cm^-1^) to the aromatic C=C absorption (1608 cm^-1^) signals for both polymerized and unpolymerized samples (n = 8). The DC was calculated according to the following formula:

DC (%) = (1: [R-cured/R-uncured]) x 100

where “R” is the ratio of aliphatic and aromatic peak intensities at 1639 cm^-1^ and 1609 cm^-1^ in cured and uncured adhesives, respectively.^26^ In addition, the more intense peaks observed for all materials and the corresponding chemical bonding were recorded. All these procedures were performed by a technician responsible for the micro-Raman device, who was blinded to all adhesive procedures.

### Statistical Analysis

The average μSBS of all resin-enamel specimens showing A/M failure mode from the same enamel specimens and tooth were obtained for statistical purposes. Specimens with cohesive and pre-test failures were not included in the data analysis. The same procedure was performed for the DC measurements. Therefore, the experimental unit of this study was the enamel specimen.

The Kolmogorov-Smirnov test was employed to assess the data distribution for normality. Barlett’s test was performed to determine the validity of the assumption of equal variances. The μSBS data were analyzed using three-way ANOVA (enamel surface vs adhesive vs enamel treatment). The in situ DC data were analyzed using two-way ANOVA (enamel surface vs enamel treatment) for each adhesive. Tukey’s test at α = 0.05 was used as post-hoc test for μSBS and in situ DC data. The enamel-etching pattern was evaluated qualitatively.

## Results

### Microshear Bond Strength

The majority of the specimens (96.8–100%) showed A/M failures (Table 2). The triple cross-product interaction was not statistically significant (enamel surface vs adhesive vs enamel treatment; p = 0.47), nor was the two double cross-product interaction (enamel surface vs enamel treatment and adhesive vs enamel treatment; p = 0.32 and p = 0.37, respectively). However, the μSBS data for the double cross-product interaction enamel surface vs adhesive was statistically significant (p = 0.0001; Table 3). Also, the main factor enamel treatment was not significant (p = 0.15; Table 3).

**Table 2:** Number of specimens (%) according to fracture mode for all experimental groups

Adhesives	Enamel treatment	Sound enamel				Fluorotic enamel			
		A/M	CE	CR	PF	A/M	CE	CR	PF
CUB	PA mode	198 (100)	0 (0)	0 (0)	0 (0)	170 (100)	0 (0)	(0)	0 (0)
	EDTA mode	200 (100)	0 (0)	0 (0)	0 (0)	180 (100)	0 (0)	0 (0)	0 (0)
FBU	PA mode	168 (98)	0 (0)	3 (2)	0 (0)	196 (96)	0 (0)	0 (0)	8 (4)
	EDTA mode	210 (100)	0 (0)	0 (0)	0 (0)	171 (98)	0 (0)	0 (0)	3 (2)
IBU	PA mode	224 (100)	0 (0)	0 (0)	0 (0)	172 (96)	4 (2)	0 (0)	4 (2)
	EDTA mode	196 (98)	0 (0)	4 (2)	0 (0)	192 (96)	0 (0)	0 (0)	8 (4)
SBU	PA mode	206 (100)	0 (0)	0 (0)	0 (0)	197 (100)	0 (0)	0 (0)	0 (0)
	EDTA mode	202 (96)	0 (0)	4 (2)	4 (2)	198 (98)	4 (2)	0 (0)	0 (0)

A/M: adhesive/mixed fracture mode; CE: cohesive-enamel fracture mode; CR: cohesive-resin fracture mode; PF: premature failure; CUB: Clearfil Universal Bond; FBU: Futurabond U; IBU: iBond Universal; SBU: Scotchbond Universal Adhesive; PA: phosphoric acid; EDTA mode: ethylenediaminetetraacetic acid.

**Table 3:** Microshear bond strength (µSBS in MPa) values (means ± standard deviations) of the universal adhesives

Adhesives	Sound enamel		Fluorotic enamel	
	PA mode	EDTA mode	PA mode	EDTA mode
CUB	20.48 ±1.2^Aa^	20.42 ± 1.2^Aa^	15.61 ± 1.5^Bb^	15.90 ± 2.0^Bb^
FBU	17.04 ± 1.5^Ab^	16.08 ± 1.5^ABbc^	13.90 ± 0.9^Cc^	13.69 ± 0.5^Cc^
IBU	15.95 ± 1.9^Ac^	16.19 ± 1.0^Abc^	11.26 ± 1.0^Cd^	12.18 ± 1.0^Cd^
SBU	18.88 ± 1.2^Aa^	18.04 ± 1.1^ABab^	16.47 ± 1.2^Ba^	16.95 ± 0.7^Ba^

CUB: Clearfil Universal Bond; FBU: Futurabond U; IBU: iBond Universal; SBU: Scotchbond Universal Adhesive; PA: phosphoric acid; EDTA mode: ethylenediaminetetraacetic acid. For each line (adhesive), different capital letters means differences statistically significant between main factors enamel surface (types of enamel and types of conditioning) (Two-way ANOVA; Tukey test, p < 0.05). For each column (enamel surface and enamel treatment) different lowercase letters means differences statistically significant between main adhesive system (Two-way ANOVA; Tukey test, p < 0.05).

When sound and fluorotic enamel were compared, a higher mean μSBS was obtained in sound enamel for all adhesives and enamel treatments (p = 0.0002; Table 3). Regarding adhesives, CUB and SBU showed statistically higher mean μSBS than did FBU and IBU in sound and fluorotic enamel (p = 0.0002; Table 3). When CUB and SBU were compared, SBU showed higher μSBS in fluorotic enamel (p = 0.001; Table 3). For pre-conditioning with EDTA, the mean μSBS were statistically similar to those obtained with the respective PA application in sound and fluorotic enamel (p = 0.15; Table 3).

### Enamel-etching Pattern

**Fig 1:**
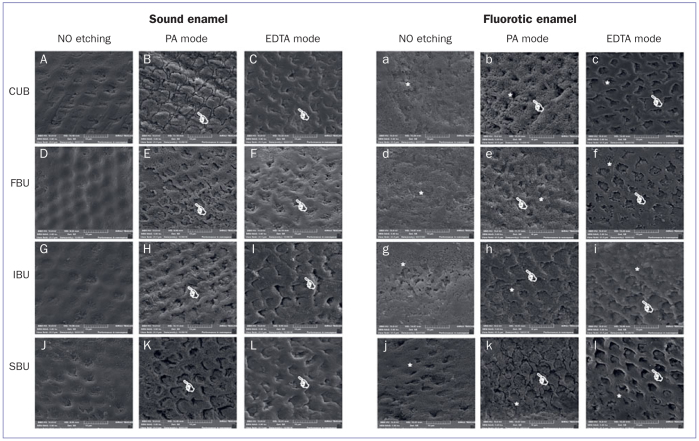
Representative SEM photomicrographs of different experimental groups tested in this study. More irregular and porous enamel surfaces are observed in fluorotic vs sound enamel (*lowercase letters, a–l). PA and EDTA completely removed the smear layer of the surface and promoted a greater dissolution of the prisms of sound enamel (white hand, capital letters) and fluorotic enamel (white hand, lower case letters). PA: phosphoric acid; EDTA: ethylenediaminetetraacetic acid.

SEM images of the enamel surfaces under the different experimental conditions are shown in Fig 1. Overall, when sound and fluorotic enamel were compared, there was more micro-irregularity and porosity over the entire fluorotic enamel surface.

Independent of the enamel surface, PA and EDTA application completely removed the smear layer from both sound and fluorotic enamel surfaces, promoting the deepest and most pronounced etching pattern when compared with no etching (Fig 1).

### In Situ Degree of Conversion

The DC values are shown in Table 4. The double cross-product interaction was not statistically significant (enamel surface vs enamel treatment; p = 0.32), as well as the main factor enamel treatment (p = 0.28; Table 4). However, the main factor enamel surface was significant (p = 0.0001; Table 4).

**Table 4:** In situ DC (%) values (means ± standard deviations) of the universal adhesives

Adhesives	Sound enamel		Fluorotic enamel	
	PA mode (**)	EDTA mode	PA mode	EDTA mode
CUB	67.43 ± 3.0^A^	66.89 ± 3.1^A^	59.48 ± 3.3^B^	57.32 ± 2.9^B^
FBU	60.12 ± 2.4^a^	62.35 ± 2.3^a^	53.65 ± 1.9^b^	51.83 ± 2.3^b^
IBU	61.45 ± 2.8^A^	59.93 ± 2.9^A^	45.64 ± 3.3^B^	44.32 ± 2.6^B^
SBU	74.80 ± 1.9^a^	73.56 ± 2.7^a^	58.39 ± 2.7^b^	61.75 ± 2.9^b^

CUB: Clearfil Universal Bond; FBU: Futurabond U; IBU: iBond Universal; SBU: Scotchbond Universal Adhesive; PA: phosphoric acid; EDTA mode: ethylenediaminetetraacetic acid. For each line (adhesive), different capital and lowercase superscript letters indicate statistically significant differences bewteen groups (two-way ANOVA; Tukey’s test, p < 0.05).

A higher mean μSBS was obtained for sound enamel than for fluorotic enamel for all adhesives and enamel treatments (p = 0.0001; Table 4). For all universal adhesives, the mean DC values were similar to those obtained with the respective PA and EDTA application in sound and fluorotic enamel (p = 0.28; Table 4).

## Discussion

The results of the present study showed that sound enamel resulted in higher mean bond strengths than did fluorotic enamel. It is known that fluorotic enamel is more acid-resistant due to the presence of fluorapatite.^12,19^ Also, fluorotic enamel presents an external hypermineralized layer that is associated with a hypomineralized sub-surface,^7,24^ which prevents adequate wetting of the enamel surface due to the low energy of the surface.^57^ Both characteristics adversely affect the bonding performance of the most recent adhesives,^9,16,17,54,67^ as well as universal adhesives to fluorotic enamel.^9,54^

Clinical alternatives have been proposed to improve bonding to fluorotic enamel. For instance, Ermis et al.^17^ showed that if fluorotic enamel was ground, the superficial removal of the enamel layer exposes a sub-surface layer that is more reactive to bonding procedures, leading to better bond strength to fluorotic enamel. This has been highly recommended in cases of moderate or severe fluorosis.^16^ However, this technique removes sound tissue, which is contrary to the minimally invasive concept.

On the other hand, mainly for self-etch adhesives, the application of phosphoric acid on fluorotic enamel is highly recommended. Mildly acidic self-etch adhesives did not sufficiently demineralize the fluorotic enamel surface, causing a less retentive pattern when compared to phosphoric enamel etching.^57,67^ In the past, it has been recommended to etch fluorotic enamel for a longer period of time,^2,40^ but it seems that 30 s of phosphoric-acid etching is sufficient to improve the surface roughness of moderately fluorotic teeth.^54^ However, future studies need to be done to evaluate the effect of different etching times on the bond strength to fluorotic enamel.

As mentioned in the introduction section, as universal adhesives are essentially one-step self-etch adhesives that can be used in different adhesion strategies,^39^ phosphoric acid is also recommended to improve the bond strength to fluorotic enamel.^9,54^

In the present study the use of pre-conditioning with EDTA was evaluated. The results showed that pre-conditioning with EDTA in both sound and fluorotic enamel yielded bond strengths similar to those produced with PA application, leading to acceptance of the first null hypothesis. EDTA is known as a potent chelating agent, with four carboxylic acid groups that promote the sequestration of metal ions to dental substrates and selectively dissolve hydroxyapatite.^41^ EDTA can also remove the surface smear layer, increase the intensity of the etching pattern, and consequently increase adhesive infiltration of the surface.^28,48^

This increase in the enamel-etching pattern intensity is shown in Fig 1. PA and EDTA were capable of creating a cleaner substrate with a more retentive etching pattern compared to non-etched substrate, leading to the acceptance of the second null hypothesis. These results corroborate the results of other authors, who reported an increase in the enamel-etching pattern when EDTA was applied for 30 s.^33^ Furthermore, these benefits could be related to the active application method. When EDTA was applied actively, the EDTA molecules were able to penetrate beyond the reach of the microbrush bristles, thus increasing the dissolution of the smear layer due to the fluid dynamics of the acid on the surface.^33^

On the other hand, pre-conditioning with EDTA did not influence the in situ DC of the adhesives; thus the third null hypothesis was accepted. It is known that when universal adhesives are applied actively, as in the present study, they improve the interaction of resin monomers with prismatic and interprismatic areas, independent of the type of universal adhesive used. Active application carryies fresh resin monomers to the deeper enamel layers. This finding was previously observed in both sound^30^ and fluorotic^9^ enamel when universal adhesives were used.

In addition, active application may increase outward solvent diffusion, mainly for adhesives composed of solvents with low vapor pressures.^63^ This solvent evaporation may allow room for changes in polymer topology by reducing the intrinsic fraction of nanopores, enabling increased cross linking and improved mechanical properties of the polymer inside the enamel hybrid layer.^8^

Of the different universal adhesives compared, SBU showed the highest μSBS in fluorotic enamel. Additionally, SBU was the only adhesive used, and after pre-conditioning fluorotic enamel with EDTA, it showed efficacy similar to that on sound enamel. SBU contains a methacrylate-modified polyprenoic acid copolymer (VCP) that potentiates the chemical interaction of SBU with hydroxyapatite^50^ once it interacts with hydroxyapatite through an exchange of calcium and phosphate ions.^34,58^ Thus, we hypothesized that even if the amount of hydroxyapatite is less in fluorotic enamel, the presence of VCP was sufficient to improve the chemical and micromechanical retention.

In general, FBU and IBU showed a lower adhesive performance when compared to CUB and SBU under all experimental conditions. This could be attributed to the composition of the universal adhesives used in the present study. CUB and SBU contained a 10-methacryloyloxydecyl dihydrogen phosphate (MDP)-based adhesive, whereas the product information sheet of FBU showed that it did not contain 10-MDP.^66^ This acidic monomer is responsible for the chemical interaction with hydroxyapatite and forms a hydrolytically stable nanolayer with calcium,^68^ which increases the mechanical strength^69^ and protects against hydrolysis.^23^

IBU has a pH of 1.6, which is lower than that of the other universal adhesives evaluated in this study, and it contains 10-MDP. However, IBU is a 2-hydroxyethyl methacrylate (HEMA)-free adhesive. Although promising results have been reported for HEMA-free adhesives in terms of bond strength,^64^ they are more prone to phase separation at the interface.^30^ In addition, IBU contains acetone as the solvent. Therefore, active adhesive application could accelerate solvent evaporation and impair the efficacy of adhesive penetration at the resin-enamel interface.^59^

In the present study, the microshear bond strength test (µSBS) was used. This test is suitable for use with smaller bonding areas (around 1mm^2^), which better concentrates the stress generated when compared to macro tests, as does the microtensile bond strength (µTBS); furthermore, both tests allow make it possible to evaluate multiple test specimens from each tooth.^49^ A significant advantage to the µSBS test over the microtensile bond strength test is that in the former, it was not necessary to trim the specimens before testing. According to Armstrong et al,^5^ “Trimming is very technique sensitive and it induces additional stress as reflected in the number of specimens that fail prior to testing, especially in weaker bonds or specimens with relatively brittle behavior.” Therefore, µSBS remain an especially useful test for a substrate like enamel, which is particularly sensitive to specimen preparation effects of µTBS testing.^5^

In the present study, active pre-conditioning with EDTA before application of universal adhesives showed similar efficacy to that of PA pre-conditioning of fluorotic enamel. Therefore, it could be an alternative to enamel etching without inducing accidental dentinal etching. However, it must be mentioned that this study provides only initial results. Hence, long-term in vitro studies and clinical studies should be conducted to elucidate the actual advantages of pre-conditioning fluorotic enamel with EDTA.

## Conclusion

Active pre-conditioning with EDTA could be considered an alternative to phosphoric acid, because the bond strength of universal adhesives to fluorotic enamel was similar for both conditioners, without compromising the other properties evaluated.

## Clinical relevance:

Active pre-conditioning with ethylenediaminetetraacetic acid (EDTA) can be considered an alternative to phosphoric acid to improve the bonding of universal adhesives to sound and fluorotic enamel.
